# Supplementation of fresh ucche (*Momordica charantia* L. var. muricata Willd) prevented oxidative stress, fibrosis and hepatic damage in CCl_4_ treated rats

**DOI:** 10.1186/s12906-015-0636-1

**Published:** 2015-04-11

**Authors:** Abu Taher Sagor, Mohammed Riaz Hasan Chowdhury, Nabila Tabassum, Hemayet Hossain, Md Mahbubur Rahman, Md Ashraful Alam

**Affiliations:** Department of Pharmaceutical Sciences, North South University, Dhaka, Bangladesh; BCSIR Laboratories, Bangladesh Council of Scientific and Industrial Research (BCSIR), Dhaka, Bangladesh

**Keywords:** *Momordica charantia*, Fibrosis, Inflammation, Reactive oxygen species, Oxidative stress, Aminotransferases

## Abstract

**Background:**

Ucche (*Momordica charantia* L. var. muricata (Willd.) Chakravarty) has been reported to possess many benefits and medicinal properties. However, the protective effect of ucche against carbon tetrachloride (CCl_4_) induced hepatotoxicity have not been clarified fully yet. The aim of the present study was to investigate the effects of ucche on oxidative stress and inflammation in liver of CCl_4_ treated rats.

**Methods:**

Female Long Evans rats were administered with CCl_4_ orally (1 ml/kg) twice a week for 2 weeks and were supplemented with freshly prepared crashed ucche (10% wt/wt of diet) with powdered chaw food. Both plasma and liver tissues were analyzed for AST, ALT and ALP activities. Oxidative stress parameters were measure by determining malondialdehyde (MDA), nitric oxide (NO), advanced protein oxidation product (APOP), and reduced glutathione (GSH) concentrations and catalase activities in plasma and liver tissues. Moreover, inflammation and tissue fibrosis were confirmed by histological staining of liver tissue sections.

**Results:**

Our data suggest that ucche significantly prevented CCl_4_-induced hepatotoxicity, indicated by both diagnostic indicators of liver damage (serum transferases activities) and histopathological analysis. Moreover, CCl_4_ administration induced profound elevation of reactive oxygen species (ROS) production and oxidative stress, as evidenced by increasing lipid peroxidation level and depletion of antioxidant enzymes in liver. Fresh ucche supplementation prevented the oxidative stresses and improved antioxidant enzyme function. Furthermore, fresh ucche supplementation reduced hepatic inflammatory cell infiltration, iron deposition and fibrosis in liver of CCl_4_ treated rats.

**Conclusion:**

In conclusion, these results suggested that the inhibition of CCl_4_-induced inflammation by ucche is due at least in part to its anti-oxidant activity and its ability to modulate the inflammation and fibrosis in liver.

## Background

Liver is the main organ for nutrition metabolism and detoxifying foreign chemical agents in living animals. Other functions of liver are helping digestion, production of invaluable endogenous hormones, enzymes as well as production of glucose while fasting. However, hepatic dysfunctions like cirrhosis, steatosis, hepatitis as well as drug induced hepatic damages are increasing day by day [1]. Oxidative stress has been proposed to be increased in most cases of liver dysfunction mentioned above. Carbon tetrachloride (CCl_4_) is a well known hepatotoxin used extensively for the study hepatotoxicity in animal models. CCl_4_ stimulates bio-activation of the phase I cytochrome P450 system to form reactive metabolic trichloromethyl radicals (^•^CCl3) and peroxy trichloromethyl radicals (^•^OOCCl_3_) [2]. These peroxy radicals stimulate kupffer cells to produce ROS, such as ^•^O_2_^−^, H_2_O_2_ and ^•^OH, which thus causes lipid peroxidation and reported to induce acute and chronic tissue injuries [3,4]. Free radicals generated by CCl_4_ are thus responsible for the membrane disintegration of hepatocytes which subsequently release marker enzymes of hepatotoxicity such as aspartate transaminase (AST), alanine transaminase (ALT), alkaline phosphatase (ALP) and lactate dehydrogenase (LDH) [5]. Centrilobular necrosis and steatosis are also commonly seen in CCl_4_- induced liver toxicities [5,6]. Inflammation and fibrosis are some other early events which may also occur during the progression of the liver damage due to CCl_4_ challenge [7,8].

Bitter melon (*Momordica charantia*) is a culinary bitter vegetable commonly used in traditional dishes in Bangladesh and Indian subcontinent [9]. Bitter melon can be found in different shapes and sizes. Generally, two varieties of bitter melon are found, mainly karela (*Momordica charantia* L. var. charantia C. B. Clarke) and ucche (*Momordica charantia* L. var. muricata (Willd.) Chakravarty). Karela is 20–30 cm long, oblong with bluntly tapering ends and pale green in color, while ucche is only 6–10 cm in length and has a narrower shape with pointed ends, and a dark green surface covered with jagged, triangular “teeth” and ridges. Both varieties are also used as a traditional medicine for the treatment of diabetes and other stomach complaints in Bangladesh. Recent evidences suggest that *Momordica charantia* extract may improve insulin resistance [10] and lowers plasma lipid profile in diabetic and obese experimental animals [11,12]. *Momordica charantia* extract also improved antioxidant status and prevents oxidative stress *in vivo* [13]. Several bioactive molecules were isolated from *Momordica charantia* leaves, fruits and seeds [14-17]. Gallic acid, tannic acid, (+)-catechin, caffeic acid, *p*-coumaric, gentisic acid, chlorogenic acid, and epicatechin have been isolated from various parts of *Momordica charantia* plants [18]. Moreover, cucurbitane type triterpenoids such as charantin, kuguacins, momordicin, and karavilagenins were also isolated from *Momordica charantia* [18]. The extract also showed antioxidant activities in various experimental models [19]. However, karela variety is extensively studied in most scientific research reported to date, whereas ucche variety is less explored for any proper scientific evaluation. Thus, the current study was undertaken to evaluate the therapeutic benefit of ucche in oxidative stress, inflammation, fibrosis and liver damage induced by CCl_4_ treated rats.

## Methods

### Plant material and preparation of extracts

*Momordica charantia* fruits were purchased from a local vegetable market in Dhaka, Bangladesh. This plant has been identified by the expert Mr. Sarker Nasir Uddin, Senior Scientific Officer, of National Herberium, Mirpur, Dhaka Bangladesh and a voucher specimen was deposited (Acc No. 40566) for future reference. Fresh fruits (250 g) were taken and cut into small pieces and macerated in electrical blander without adding any water. This suspension was used as a supplementation to add with powder food (10% W/W of diet).

### Animals and treatment

Ten to twelve weeks old, 24 Long Evans female rats (150–210 g) were obtained from Animal production unit of Animal House at Department of Pharmaceutical Sciences, North South University and were kept in individual cages at room temperature of 25 ± 3°C with a 12 h dark/light cycles. They have free access to standard laboratory feed (Pellet food was crushed to powder) and water, according to the study protocol approved by Ethical Committee of Department of Pharmaceutical Sciences, North South University for animal care and experimentation. To study the hepatoprotective effects of Ucche, rats were equally divided into four groups (six rats each): Group I (Control), Group II (Control + Ucche), Group III (CCl_4_) and Group IV (CCl_4_ + Ucche). Animals of group I and II were treated with 1 ml/kg of saline (0.85%) and olive oil (1 ml/kg) intragastrically twice a week for two weeks. Rats of group II were supplanted with Ucche fruit in powder food (10% of powder food, w/w). Rats of group III and IV were treated with CCl_4_ (1:3 in olive oil) at a dose of 1 ml/kg intragastrically twice a week for two weeks. Animals of group III received only CCl_4_ treatment; however, animals of group IV received ucche fruit crashed in powder food (10% of powder food, w/w) along with CCl_4_ treatment. Animals were checked for the body weight, food and water intake on a daily basis. After 14 days all animals were weighted, sacrificed, collected the blood and all internal organs such as heart, kidney, spleen and liver. Immediately after collection of the organs, they were weighted and stored in neutral buffered formalin (pH 7.4) for histological analysis and in refrigerator at −20°C for further studies. Collected blood was centrifuged at 8000 rpm and separated the plasma and stored in refrigerator at −20°C for further analysis.

### Assessment of hepatotoxicity

Liver marker enzymes (alanine aminotransferase (ALT), aspartate aminotransferase (AST), and alkaline phosphatase (ALP) were estimated in plasma by using Diatech diagnostic kits (Hungary) according to the manufacturers protocol.

### Preparation of tissue sample for the assessment of oxidative stress markers

For determination of oxidative stress markers, liver tissue was homogenized in 10 volumes of Phosphate buffer containing (pH 7.4) and centrifuged at 12,000 × g for 30 min at 4°C. The supernatant was collected and used for the determination of protein and enzymatic studies as described below.

### Estimation of lipid peroxidation

Lipid peroxidation in liver was estimated colorimetrically measuring malondialdehyde (MDA) followed by previously described method [20]. In brief, 0.1 ml of tissue homogenate (Tris–HCl buffer, pH 7.5) was treated with 2 ml of (1:1:1 ratio) TBA-TCA-HCl reagent (thiobarbituric acid 0.37%, 0.25 N HCl and 15% TCA) and placed in hot water bath for 15 min and cooled. The absorbance of clear supernatant was measured against reference blank at 535 nm.

### Estimation of nitric oxide (NO)

Nitric oxide (NO) was determined according to the method described by Tracy et al. as nitrate and nitrite [21]. In this study, Griess-Illosvoy reagent was modified by using naphthyl ethylene diamine dihydrochloride (0.1% w/v) instead of 1-napthylamine (5%). The reaction mixture (3 mL) containing brain homogenates (2 mL) and phosphate buffer saline (0.5 mL) was incubated at 25°C for 150 min. A pink colored chromophore was formed which was measured at 540 nm against the corresponding blank solutions with a spectrophotometer. NO level was calculated as nitrate by using Sodium nitrate standard curve and expressed as nmol/mL.

### Estimation of Advanced protein oxidation products (APOP)

APOP level was performed using previously described method reported by Witko-Sarsat [22] and Tiwari [23]. Two mL of plasma was diluted (1:5) in PBS, 0.1 mL potassium iodide (1.16 M) was then added to each tube, followed by 0.2 mL acetic acid after 2 min. The absorbance of the reaction mixture was immediately read at 340 nm against a blank containing 2 mL of PBS, 0.1 mL of KI, and 0.2 mL of acetic acid. The chloramine-T absorbance at 340 nm was found linear within the range of 0 to 100 nmol/mL, APOP concentrations were expressed as nmol · mL^−1^ chloramine-T equivalents.

### Estimation of catalase (CAT) activity

CAT activities were determined using previously described method [24,25] with some modifications. The reaction solution of CAT activities contained: 2.5 ml of 50 mmol phosphate buffer (pH 5.0), 0.4 ml of 5.9 mmol H_2_O_2_ and 0.1 ml enzyme extract. Changes in absorbance of the reaction solution at 240 nm were determined after one minute. One unit of CAT activity was defined as an absorbance change of 0.01 as units/min.

### Estimation of reduced glutathione (GSH)

Reduced glutathione was estimated by the method of Jollow et al. [26]. The total volume of 3.0 ml assay mixture composed of 0.1 ml filtered aliquot, 2.7 ml phosphate buffer (0.1 M, pH 7.4) and 0.2 ml DTNB (5,5-dithiobis-2-nitrobenzoic acid), (100 mM). The yellow color of the mixture was developed, read immediately at 412 nm on a Smart SpecTM plus Spectrophotometer and expressed as ng/mg protein.

### Histopathalogical assessments

For microscopic evaluation liver tissues were fixed in neutral buffered formalin and embedded in paraffin, sectioned at 5 μm and subsequently stained with hematoxylin/eosin to see the architecture of hepatic tissue and inflammatory cell infiltration. Sirious red staining for fibrosis and prussiun blue staining for iron deposition were also done in liver sections. Sections were then studied and photographed under light microscope (Zeiss Axioscope) at 40X magnifications.

### Statistical analysis

All values are expressed as mean ± standard error of mean (SEM). The results were evaluated by using the Two way or One-way ANOVA followed by Bonferroni test using Graph Pad Prism Software, version 6. Statistical significance was considered *p* < 0.05 in all cases.

## Result

### Effect on body weight, food and water intake

**Figure 1:**
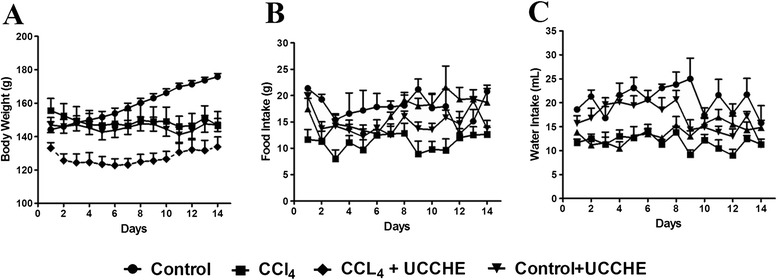
**Effect of bitter melon (Ucche) on body weight (A), food intake (B) and water intake (C) in CCl**_**4**_ **treated rats.**

Body weight of each rat was recorded every day during the experiment, and % change was calculated for all groups. It was found that the body weights of rats were decreased in CCl_4_ treated rats compared to the control (Figure 1). However, ucche supplementation did not improve the weight reduction in CCl_4_ treated rats. Moreover, CCl_4_ intoxicated group significantly decreased food and water intake compare to control rats, reduction of food and water intake was further improved in ucche treated group (Figure 1).

### Effect on organ wet weight

Table 1 shows the effect of various treatments on the rats’ organs weight. The spleen wet weight was significantly (p < 0.05) increased in the CCl_4_-treated rats compared to control group rats. Ucche (10% per kg of diet) treatment was significantly (p < 0.05) attenuated the wet weight gain of the spleen in the CCl_4_-treated rats. Moreover, CCl_4_-treated rats showed slight decrease in liver wet weight, however, ucche (10% per kg of diet) treatment did not change the wet weight of the liver compared to the control (Table 1). Another crucial finding in this study was the reduction of kidney wet weight due to CCl_4_ intoxication. Apart from these findings, CCl_4_ intoxication increased the heart wet weight in rats which was normalized due to ucche supplementation (Table 1).

**Table 1:** **Effect of bitter melon (Ucche) on body weight, food and water intake and organ weight of CCl**_**4**_ **treated rats**

**Parameters**	**Control**	**Control + Ucche**	**CCl**_**4**_	**CCl**_**4**_ **+ Ucche**	***p*** **values**		
					**CCl**_**4**_	**Treatment**	**Interaction**
**Initial Body weight**	144.15 ± 4.65	146.93 ± 4.51	155.67 ± 7.13	133.14 ± 3.19	0.0752	0.8310	0.0261
**Final Body weight**	175.83 ± 1.90	147.00 ± 3.81	146.68 ± 8.33	133.90 ± 5.89	0.0013	0.0012	0.1630
**Food intake/d**	17.8 ± 0.64	14.75 ± 0.60	11.08 ± 0.46	16.74 ± 0.81	0.0013	0.0509	<0.0001
**Water intake/d**	20.58 ± 0.77	16.94 ± 0.89	11.80 ± 0.41	13.72 ± 0.54	0.7271	0.0722	0.7751
**Liver wet weight**	6.09 ± 0.36	5.25 ± 0.10	5.62 ± 0.25	4.93 ± 0.47	<0.0001	0.2282	0.0134
**Kidney wet weight**	1.04 ± 0.07	0.95 ± 0.03	0.86 ± 0.04	0.71 ± 0.19	0.5910	<0.0001	0.9141
**Heart wet weight**	0.42 ± 0.02	0.46 ± 0.01	0.52 ± 0.01	0.45 ± 0.05	0.1995	<0.0001	0.1995
**Spleen wet weight**	0.49 ± 0.06	0.58 ± 0.02	0.81 ± 0.09	0.58 ± 0.04	<0.0001	0.3009	0.0067

Values are presented as mean ± SEM. N = 5-7 in each group or otherwise specified. Two way or One way ANOVA with Bonferoni tests were done as post hoc test. Values are considered significance at p < 0.05. a vs b, control vs CCl_4_; b vs c, CCl_4_ vs ucche treatment.

### Effect on biochemical parameter of liver functions

Biochemical measurement of liver functions revealed that, CCl_4_ induced a significant increase in plasma AST, ALT, and ALP activity compared with control values, respectively (Table 2). Treatment of animals with ucche (10% per kg of diet) concomitantly with CCl_4_ significantly counteracted the alteration in all hepatotoxicity indices compared to the CCl_4_-intoxicated group. In addition, treatment of animals with ucche alone for 2 weeks did not show any significant change in liver enzymes compared with the control group (Table 2).

**Table 2:** **Effect of bitter melon (ucche) on biochemical parameter in plasma and liver**

**Parameters**	**Groups**				***p*** **values**		
	**Control**	**Control + Ucche**	**CCl**_**4**_	**CCl**_**4**_ **+ Ucche**	**CCl**_**4**_	**Treatment**	**Interaction**
**Plasma**							
**AST(U/L)**	19.0 ± 1.6	21.32 ± 1.68	37.9 ± 3.1	23.3 ± 4.0	0.0374	0.0012	0.0062
**ALT(U/L)**	13.8 ± 1.7	16.59 ± 2.62	30.5 ± 3.1	18.7 ± 1.9	0.0831	0.0012	0.0082
**ALP(U/L)**	50.8 ± 3.3	50.92 ± 2.76	81.2 ± 6.1	63.9 ± 4.7	0.0731	0.0001	0.0690
**MDA(nmol/mL)**	6.34 ± 0.37	3.57 ± 0.54	7.7 ± 0.91	6.71 ± 0.77	0.0731	0.0001	0.0690
**NO (nmol/mL)**	3.22 ± 0.27	2.24 ± 0.22	5.8 ± 0.3	1.15 ± 0.32	<0.0001	0.0195	<0.0001
**APOP** (nmol/mL equivalent to Chloramine-T)	82.60 ± 8.49	67.0 ± 9.2	164.4 ± 15.15	84.3 ± 8.8	0.0340	0.0102	0.3072
**Catalase (U/min)**	6.00 ± 0.32	4.28 ± 0.38	2.93 ± 0.60	5.07 ± 0.74	0.6960	0.0343	0.0011
**GSH (ng/mg protein)**	20.05 ± 1.81	18.39 ± 1.03	12.72 ± 0.57	16.39 ± 0.57	0.3788	0.0005	0.0270
**Liver**							
**AST(U/L)**	36.18 ± 3.22	31.01 ± 3.46	65.18 ± 3.87	46.51 ± 2.11	0.0023	<0.0001	0.0594
**ALT(U/L)**	26.00 ± 3.88	34.67 ± 2.24	77.61 ± 4.80	28.77 ± 1.49	<0.0001	<0.0001	<0.0001
**ALP(U/L)**	30.33 ± 3.67	31.42 ± 3.54	113.53 ± 3.71	68.25 ± 3.67	<0.0001	<0.0001	<0.0001
**NO (nmol/mL)**	15.87 ± 1.56	20.21 ± 0.45	35.62 ± 4.21	18.11 ± 2.17	0.0225	0.0036	0.0006
**MDA (nmol/mL)**	21.85 ± 1.86	24.62 ± 4.88	57.16 ± 6.44	36.92 ± 1.98	0.0741	<0.0001	0.0226
**APOP** (nmol/mL equivalent to Chloramine-T)	365.00 ± 40.19	450.0 ± 75.83	1357.62 ± 91.06	977.50 ± 63.05	0.0491	< 0.0001	0.0097
**Catalase (U/min)**	52.13 ± 4.02	68.40 ± 5.92	26.32 ± 3.82	50.27 ± 2.33	0.0003	< 0.0001	0.3967
**GSH (ng/mg protein)**	21.22 ± 1.91	19.22 ± 1.13	45.98 ± 5.05	32.89 ± 1.79	0.0171	< 0.0001	0.0703

Values are presented as mean ± SEM. N = 5-6 in each group or otherwise specified. Two way or One way ANOVA with Bonferoni tests were done as post hoc test. Values are considered significance at p < 0.05. a vs b, control vs CCl_4_; b vs c, CCl_4_ vs ucche treatment. APOP-Advanced protein oxidation product, expressed as nmol/mL equivalent to Chloramine-T.

### Oxidative stress markers and antioxidant enzymes

To determine the oxidative stress in our study, we evaluated the MDA, nitric oxide and APOP content in plasma and liver homogenates. CCl_4_ induced rats showed a higher concentration of lipid peroxidation product MDA both in plasma and liver homogenates (Table 2) (7.7 ± 0.91 and 57.16 ± 6.44 nmol/mL in plasma and liver homogenates respectively). Additionally, ucche (10% per kg of diet) co-treatment significantly reduced the level of lipid peroxides compared to CCl_4_ intoxicated group (6.71 ± 0.77 and 36.92 ± 1.98 nmol/mL in plasma and liver homogenates respectively); however, lipid peroxide level was still higher than the control.

CCl_4_ have profound effect on APOP development in plasma and liver. CCl_4_ challenge significantly increased the APOP concentration in plasma and liver (164.4 ± 15.15 and 1357.62 ± 91.06 nmol/mL equivalent to Chloramine-T respectively) which was decreased due to ucche (10% per kg of diet) supplementation in CCl_4_ intoxicated rats (84.3 ± 8.8 and 977.50 ± 63.05 nmol/mL equivalent to Chloramine-T respectively).

Nitric oxide measured as nitrate was also increased both in plasma and liver homogenates (5.8 ± 0.3 and 35.62 ± 4.21 nmol/mL in plasma and liver homogenates respectively) compared to control rats which was normalized by ramipril treatment in CCl_4_ intoxicated group (Table 2). CCl_4_ induced a significant decrease in liver antioxidant enzyme activities GSH and CAT respectively, compared to the control levels. In addition, CCl_4_ induced a significant increase in lipid peroxide level by more than four folds compared with the control group (Table 2). Treatment of animals with ucche (10% per kg of diet) concomitantly to CCl_4_ significantly counteracted the oxidative stress effect of CCl_4_. It was found that, the level of GSH and CAT activity restored near normal compared to CCl_4_ intoxicated group (Table 2).

### Inflammation, fibrosis iron deposition in liver

**Figure 2:**
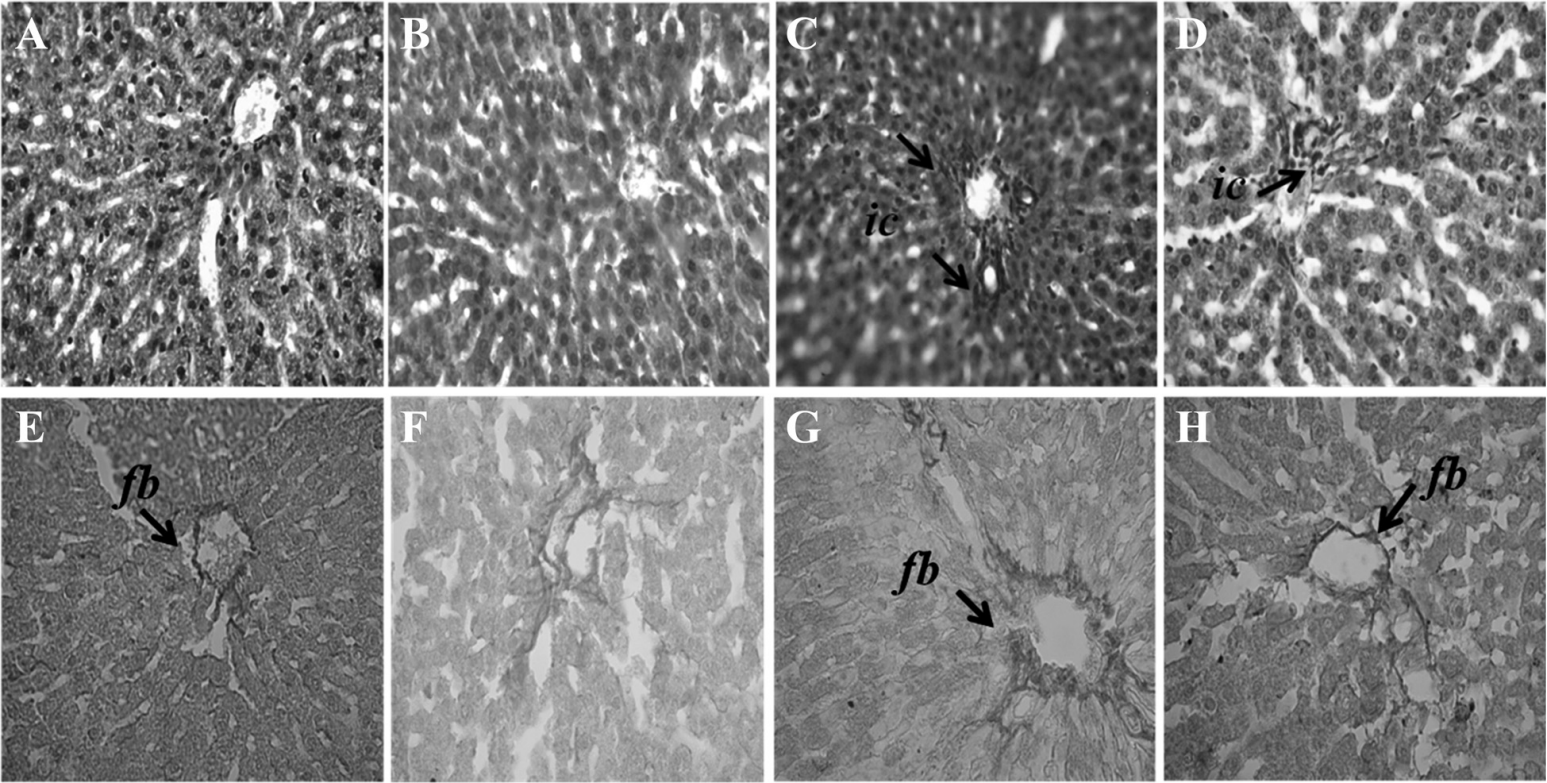
**Effect of bitter melon (Ucche) on hepatic inflammation and fibrosis in CCl**_**4**_ **treated rats. A, E**- Control; **B, F**- Control + Ucche; **C, G**- CCl_4_ and **D, H**- CCl_4_+ Ucche. Magnification 40x. *ic*-inflammatory cells; *fb*- fibrosis.

**Figure 3:**
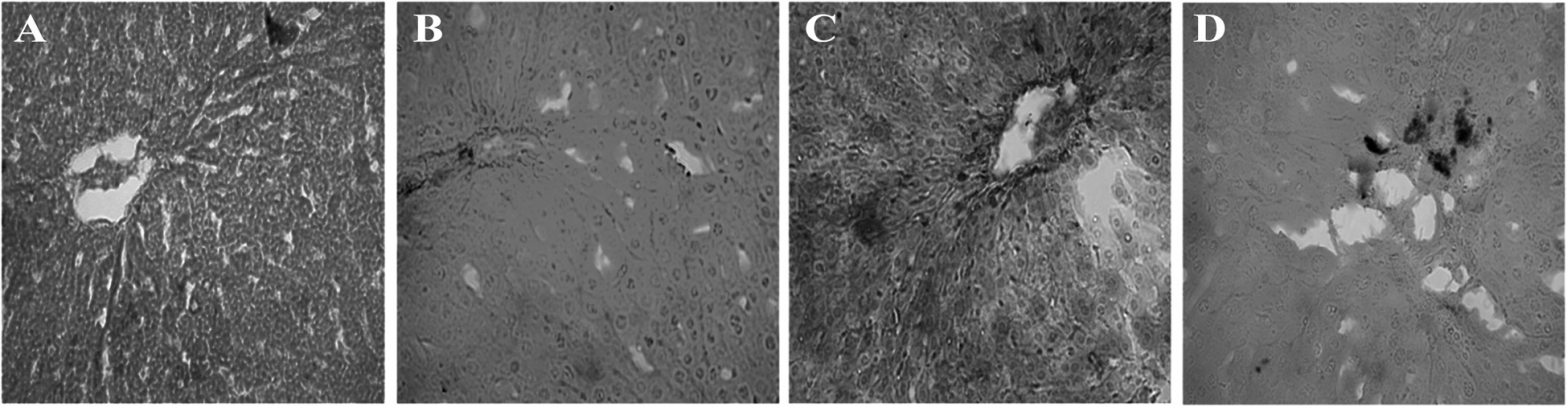
**Effect of ucche on hepatic iron deposition in CCl**_**4**_ **treated rats. A**- Control; **B**- Control + Ucche; **C**- CCl_4_ and **D**- CCl_4_+ Ucche. Blue dots- iron pigment, Magnification 40x.

Inflammation was seen in rats treated with CCl_4_. Massive serge of inflammatory cells was found in the centilobular part of liver section stained with H & E staining in CCl_4_ treated rats group (Figure 2C). Necrotized tissue scar and ballooning of the hepatocytes were also seen in liver of CCl_4_ treated rats. ucche (10% per kg of diet) co-treatment attenuated the inflammatory cell infiltration and necrosis in the liver tissues of CCl_4_ treated rats (Figure 2D). Liver fibrosis was evaluated by histologically by visualizing the red color of collagen fibers using Sirius red stain. In contrast, the collagen fibers were heavily deposited around portal tracts and central veins in CCl_4_-intoxicated group and extended from central vein to portal tract resulting in the formation of pseudolobules (Figure 2G). Histological staining also revealed massive iron deposition in liver section stained for free iron depot in CCl_4_ treated rats (Figure 3C). Ucche supplementation decreased this iron deposition in CCl_4_ treated rats (Figure 3D).

## Discussion

Liver is one of the vital organs in our body responsible for detoxification of toxic chemicals and drugs. Chronic liver diseases are increasing in recent years and the medical treatments for these diseases are usually difficult to handle and showed limited efficacy. Therefore, it is necessary to find new alternative treatment for the management of liver diseases. CCl_4_ treatment is one of the most used methods for the study of experimental hepatic dysfunction nowadays; however, CCl_4_ is considered as an extremely toxic chemical agent [27]. Several important basic mechanisms involving of tissue damages induced by CCl_4_ have been proposed, such as reactive free radical metabolites, lipid peroxidation, metabolic activation, covalent binding and disturbance of calcium homeostasis [3]. The present study demonstrates that fresh ucche supplementation prevented oxidative stress, lowered inflammatory cell infiltration and fibrosis in liver and improved the hepatic enzymes function against CCl_4_-induced liver injury in rats.

Oxidative stress is the major cause of tissue damage in CCl_4_ treated animal model of hepatic dysfunction. CCl_4_ is metabolized by liver cytochrome P450 and converted into the free radical ^•^CCl_3_ [28,29]. The free radical attacks hepatocytes and causes necrosis of parenchymal cells. We found that the concentration of MDA and APOP, which are indices of oxidative stress, were elevated in CCl_4_-challenged rats. However, decreased concentrations of these two compounds were found in plasma and the liver tissues of those CCl_4_-rats that were treated with ucche supplementation, which suggests that fresh ucche can effectively protect against lipid peroxidation and protein oxidation. Similar results were also reported by other investigators that fruit extract (300 mg/kg) and the plant extract at a dose of 5 ml/kg also produced significant protection of liver damage by preventing elevated TBARS and hydroperoxides concentration and increased the levels of glutathione peroxidase (GPx), superoxide dismutase (SOD), catalase and reduced glutathione in ammonium chloride-induced toxicity in rats [13,30]. Catalase is an important antioxidant enzymes responsible for the scavenging of free radical generated in tissue. Catalase enzyme exists in all aerobic cells, is a hemeprotein that metabolizes the H_2_O_2_ to form oxygen and water. Szymonik-Lesiuk *et al*. reported that CCl_4_ intoxication leads to changes in antioxidant enzymes activity [31]. In our study, CCl_4_ treatment causes breakdown of catalase mediated prevention of tissue damage and lowered the catalase activity which was further improved to near normal by ucche supplementation. Oxidative stress mediated tissue damage also increases the liver marker enzyme activities. Damage to the liver typically results in leakage of AST and ALT into the bloodstream [32]. In the present study CCl_4_ administration to rats caused a significant increase in serum AST, ALT and ALP activity which were further normalized by ucche supplementation. Nitric oxide is another mediator of oxidative stress despite of its valuable role in the maintenance of vascular tone and as signaling molecule. However, excess production of nitric oxide by iNOS may lead to hepatic injury which can be reversed by scavenging of nitric oxide [33,34]. Ucche supplementation also reduces the elevated nitrate concentration in plasma and tissues of CCl_4_ treated rats. Our results are also comparable to Chaudhari et al. who reported that hydroalcoholic extract of the plant leaves (100 and 200 mg/kg) normalized the levels of SGOT, SGPT, ALP, total bilirubin and prevented steatosis, centrilobular necrosis and vacuolization in liver of carbon tetrachloride induced liver damage in rats [35]. Similar findings were also reported in another study that investigated the hepatoprotective effects of plant bioactive compounds against CCl_4_-induced injury in rats hepatocytes [36].

Moreover, oxidative stress mediated tissue damage may trigger inflammation and fibrosis in liver of CCl_4_ treated rats. Liver fibrosis is also characterized by a distortion of liver tissue architecture and an excessive deposition of extra cellular matrix (ECM) proteins that type I collagen predominates [7]. Hepatic stellate cells (HSCs) have been recognized as the main matrix-producing cells in the process of liver fibrosis. Kupffer cells (KCs) could activate HSCs by producing and releasing a variety of mediators, such as proinflammatory cytokines and reactive oxygen species that fulfill a crucial function in the inflammatory responses of the liver [37]. Histological examination also demonstrated that a large number of inflammatory cells infiltrated into the intralobular and interlobular regions together with increased collagen fibers in CCl_4_ treated rats compared with normal rats. On the contrary, ucche supplementation remarkably reduced the immigration of inflammatory cells and the deposition of collagen fibers compared with rats in model group treated with CCl_4_.

Another key finding in our model is the deposition of iron in hepatic tissues assessed through histologically by iron staining. Iron deposition is characteristics of many forms of liver diseases including viral hepatitis-C, alcoholic and nonalcoholic hepatitis [38,39]. In hepatic diseases, the Kupffer cells (KC) and liver cells are not capable of processing the iron due to heam oxygenase deficiency and deposited in the tissue which further contributes to oxidative tissue damage by fenton reaction [40-42]. Previous authors also reported that iron overload can enhance the development of liver cirrhosis in animal models [43,44]. Clinical evidence also noted that iron-loaded livers of hemochromatosis patients were also found to exhibit oxidative stress and enhanced expression of TGF-β1 [45]. TGF-β1 is the key regulatory growth factor secreted by fibroblast cells due to activation by inflammatory stimuli and oxidative stress [46]. Thus, the deposited iron can also accelerate the advancement of liver fibrosis by increasing α1-(I)-collagen mRNA expression [47]. In our study, ucche supplementation significantly reduced the iron deposition and fibrosis in liver of rats treated with CCl_4_ further confirmed the reduction of oxidative stress in liver.

Phenolic or polyphenolic compounds are potent antioxidants and are capable of preventing oxidative stress in various diseases including liver damage [48]. Previous reports suggest that various phenolic compounds such as gallic acid, tannic acid, (+)-catechin, caffeic acid, *p*-coumaric, gentisic acid, chlorogenic acid, and epicatechin are present in *Momordica charantia* plants [14,15]. Our analysis (unpublished data) on ucche fruit extract showed that ellagic acid and myricetin are present in abundant amount. Ellagic acid showed preventive effect in isoniazid-rifampicin induced liver damage [49] and cisplatin-induced oxidative stress in liver [50]. Myricetin are also effective in lowering plasma lipids (which is one of the mechanism of hepatic protection in non alcoholic fatty liver like diseases), in high fat diet fed animals [51]. However, it is not possible to speculate which phenolic compound responsible for the preventive activity in liver showed by ucche supplementation in this study, probably a combined effects of all phenolic compounds play their role and gave a synergistic effect.

## Conclusions

In conclusion, our findings revealed that ucche supplementation has a protective effect against CCl_4_ induced hepatotoxicity. The hepatoprotective effects of ucche supplementation are likely related to the prevention of oxidative stress by increasing antioxidant activity, lowering lipid peroxidation, and improving the function of liver cells. Moreover, ucche supplementation also promotes hepatic protection by decreasing inflammation and fibrosis probably mediated by inhibiting iron mediated signaling pathways and HSCs activation. Further studies are needed to investigate the molecular mechanism of the hepatoprotective effect of ucche supplementation in CCl_4_ treated rats.
